# Delta-opioid receptor–mediated neural protection and regeneration

**DOI:** 10.4103/NRR.NRR-D-25-00293

**Published:** 2025-09-29

**Authors:** Jiahui Li, Yuan Xu, Ziyu Chao, Leena Khiati, Ying Xia

**Affiliations:** 1 Shanghai Key Laboratory of Acupuncture Mechanism and Acupoint Function, Fudan University, Shanghai, China; 2 Shanghai Research Center for Acupuncture and Meridians, Shanghai, China; 3 Department of Neurosurgery, The First People’s Hospital of Changzhou, Changzhou, Jiangsu Province, China; 4 Clinical Medical Research Center, The Third Affiliated Hospital of Soochow University, Changzhou, Jiangsu Province, China; 5 Dubai Medical University, College of Medicine, Dubai, The United Arab Emirates

**Keywords:** Alzheimer’s disease, apoptosis, brain-derived neurotrophic factor, delta-opioid receptor, hypoxia-ischemia, ischemic stroke, nerve regeneration, neuroinflammation, neuroprotection, Parkinson’s disease

## Abstract

The delta-opioid receptor was previously viewed as a mediator in pain regulation. Recent data shed light on its specific role in neural protection and regeneration. An up-regulation of delta-opioid receptor expression and/or activity protects neuronal cells/tissues against various injuries and promotes neural regeneration. This review focuses on these new findings and the underlying mechanisms. In particular, we summarize the following key points: (1) the role of delta-opioid receptor in neuroprotection across various models and conditions; (2) the mechanisms of delta-opioid receptor neuroprotection against acute injury; (3) the neuroprotective mechanisms of delta-opioid receptor during prolonged injury; (4) delta-opioid receptor protection against ischemic and degenerative brain diseases and the underlying mechanisms; and (5) the regulation of delta-opioid receptor in neural regeneration. This article aims to provide an overview of delta-opioid receptor-mediated neural protection and regeneration, as well as its potential in treating neurological diseases.

## Introduction

The incidence of nervous system diseases, especially ischemic brain injury and neurodegenerative diseases (such as ischemic stroke, Alzheimer’s disease [AD], Parkinson’s disease [PD], amyotrophic lateral sclerosis) is increasing year by year worldwide, causing a significant health burden on society (GBD 2021 Nervous System Disorders Collaborators, 2024; Li et al., 2024). Ischemic and degenerative brain disorders have become common causes of disability and death among the elderly. Their incidence increases with age, particularly among individuals over 60 years old (Jiang et al., 2025).

Although some treatment options are available, many neurological diseases still lack effective therapies, and most existing treatments focus on relieving symptoms (Rashad et al., 2022; Jucker and Walker, 2023; Foltynie et al., 2024). Therefore, the importance of neuroprotection and neural regeneration research has become increasingly prominent. Neuroprotection focuses on delaying or preventing the process of nerve cell death (Hasler and Inta, 2024), while neural regeneration is dedicated to repairing damaged nerve tissue and rebuilding its functional integrity (Saraswathy et al., 2024). These two research directions complement each other and together constitute a key path to breaking through the current treatment bottleneck.

In recent years, an increasing number of studies have shown that the delta-opioid receptor (DOR) plays a crucial role in neuroprotection and neural regeneration. Our studies and those of others have consistently shown that activating DOR inhibits neuronal apoptosis, reduces inflammation, and promotes nerve regeneration (Ma et al., 2005; Narita et al., 2006; Hong et al., 2007; Georganta et al., 2013; Tian et al., 2013; Wang et al., 2014; Xu et al., 2025; Qiu et al., 2019; Zhang et al., 2022b), shedding light on new hope for better prevention/treatment of neurological diseases.

The aim of this review is to comprehensively outline the neural protection and regeneration induced by DOR in different models, delve into the underlying mechanisms, and consider potential clinical uses of DOR as a new molecular target for treating neurological disorders.

## Search Strategy

In this narrative review, we searched the literature from 1991 to 2025 using PubMed. The following keywords were used: “delta opioid receptor” and “neuroprotection,” or “neurodegenerative disease” or “ischemic stroke” or “hypoxic injury” or “neural differentiation.” The papers considered most relevant to the focus of this review were included. A preliminary screening was conducted by reading the titles and abstracts of the retrieved studies to determine the relevant research for this review. After that, we read the full text and summarize the comment.

## Opioid Receptors in Neuroprotection

The opioid receptor family mainly comprises three key members: DOR, kappa-opioid receptor (KOR), and mu-opioid receptor (MOR) (Jelen et al., 2022). They belong to G protein-coupled receptors and are distributed widely in the peripheral and central nervous system (CNS) with diverse functions (Mansour et al., 1994).

DOR is widely distributed in the cortex, caudate putamen, and amygdala of the brain (Xia and Haddad, 1991, 2001). A previous study suggests that DOR has properties of relieving chronic pain, but it is less important than MOR (Vicario et al., 2022). Interestingly, we found that the density of DOR in turtle brains, which are more resistant to ischemia and hypoxia compared to rat brains, is significantly higher in turtles than in rats (Xia et al., 1992; Xia and Haddad, 2001). This phenomenon prompted us to explore the potential role of DOR in neuroprotection against hypoxic and ischemic insults. Indeed, we first found the unique role of DOR in neuroprotection against hypoxic/excitotoxic injury (Zhang et al., 2000, 2002). In the following decades, we and other global researchers conducted serial studies that demonstrated the significance of DOR as a key target for neuroprotection under various conditions, such as hypoxic, ischemic and excitotoxic stress, neuroinflammation, neurodegenerative injury, and neurodegenerative diseases (Mayfield et al., 1996; Zhang et al., 2000, 2002, 2006; Lim et al., 2004; Ma et al., 2005; Zhao et al., 2005; Chao et al., 2007a, b, 2008; Iwata et al., 2007b; Su et al., 2007; Xiong et al., 2007; Charron et al., 2008; Govindaswami et al., 2008; Pamenter and Buck, 2008; Zhu et al., 2009; Gao et al., 2010, 2012; Turner and Johnson, 2011; Kim et al., 2012; Tian et al., 2013; Zhou et al., 2013; Wang et al., 2014; Liu et al., 2015). The activation of DOR using both exogenous and endogenous agonists has been shown to enhance nerve cell survival and reduce cell death. Conversely, DOR antagonists have been found to inhibit the protective effects of DOR (Zhang et al., 2006; Wang et al., 2011, 2016). The neuroprotective mechanisms of DOR involve multiple levels of regulation, including ion homeostasis, enhancement of pro-survival signaling pathways, inhibition of cell apoptosis pathways, control of inflammatory signals, microRNA, autophagy, mitochondria, microglia, and neural regeneration.

There have been studies on MOR and KOR regarding their roles in neural protection, although there are still some controversies. MOR is the most widely studied opioid receptor in the past because of its strong function in pain relief (Zhang et al., 2022a). We previously noticed that MOR activation had no neuroprotective effect (Zhang et al., 2000, 2002; Chao et al., 2007a). Some studies suggested that MOR agonist, morphine, might exert a neuroprotective effect via DOR, not MOR (Lim et al., 2004; Gwak et al., 2010; Ryu et al., 2018). In contrast, previous studies have shown that MOR is a negative regulatory factor in neuroprotection (Myers et al., 2023; Rodriguez et al., 2024). In a mouse model of traumatic brain injury, MOR expression increased in the neocortex compared to the sham group. Naltrexone, a MOR antagonist, improved neurodegenerative injury and inhibited seizures (Rodriguez et al., 2024).

KOR is a therapeutic target for treating pain, and relieving addiction and affective disorders (Han et al., 2023). Our early work showed that KOR provided mild protection to neurons against injury (Zhang et al., 2000, 2002). A later study also suggested that KOR is a neuroprotective factor. KOR activation protected against brain damage in ischemic and reperfusion injury in rats (Fang et al., 2013). Injecting synthetic Dynorphin A into the lateral ventricle improved behavioral disorders, reduced the volume of cerebral infarction, and decreased neuronal cell apoptosis in middle cerebral artery occlusion (MCAO) rats. The protective effect of dynorphin A was reversed by KOR inhibitors, suggesting that dynorphin exerted neuroprotection through KOR (Chen et al., 2021b).

Due to the extensive databases and conclusive findings regarding DOR-mediated neural protection and regeneration, the following sections will concentrate on the evolution of research from past to present in relation to DOR as a therapeutic target for alleviating various stressors and associated mechanisms, as well as the potential protective role of DOR in neurodegenerative diseases.

## Delta-Opioid Receptor Neuroprotection

Substantial evidence in *in vitro* and *in vivo* studies has verified that DOR has a neuroprotective role under various insults. This section summarizes the neuroprotection of DOR in cell and animal models following hypoxic, ischemic, and excitotoxic stress and the protection for neurodegenerative diseases.

### Delta-opioid receptor protection *in vitro* models

*In vitro* studies with neuronal cell lines and primary neurons consistently show that DOR displays a neuroprotective action.

#### Delta-opioid receptor neuroprotection against hypoxic injury

We provided the first evidence showing that DOR activation is neuroprotective (Zhang et al., 2000, 2002). In cultured cortical neurons, DOR activation with [D-Ala2, D-Leu5] enkephalin (DADLE), a synthetic DOR agonist, reduced neuronal damage induced by hypoxic exposure. In contrast, neurons treated with naltrindole, a DOR antagonist, aggravated hypoxic injury (Zhang et al., 2002). Kim et al. (2012) also observed that exposure to 24-hour oxygen deprivation (OD) reduced neuronal survival, while DOR activation by DADLE protected the neurons from OD. Similarly, DOR activation using UFP-512, a specific DOR agonist, protected PC12 cells against hypoxic injury (Wang et al., 2014).

Interestingly, we found that mild and transient hypoxia can induce a protective effect on the neurons, called hypoxic preconditioning (HPC). The prior-HPC-treated neurons are more resistant to subsequent severe hypoxic insult (Ma et al., 2005; Zhang et al., 2006; Zhao et al., 2006). Our previous study showed that although severe hypoxia led to decreased DOR expression in cortical neurons, a delayed HPC increased the mRNA and protein levels of DOR in the neurons, a likely mechanism for HPC neuroprotection (Ma et al., 2005). Conversely, the DOR antagonists abolished the HPC protection effect, further supporting that HPC-induced neuroprotection depends on DOR function (Ma et al., 2005).

DOR protection is involved in the regulation of astrocytes, with a notable increase in DOR expression in astrocytes after 6 hours of hypoxia (Wang et al., 2014). DOR activation with UFP-512 reversed the decrease in cell viability by hypoxic insult (Wang et al., 2014). Meanwhile, adding DOR antagonist naltrindole to the medium reversed the protective effect on the astrocytes induced by DOR activation (Wang et al., 2014).

#### Delta-opioid receptor neuroprotection against ischemic injury

Substantial data from *in vitro* models confirmed the neuroprotective effect of DOR on oxygen-glucose deprivation (OGD)-induced neuronal injury. Morphine preconditioning of rat brain slices prior to OGD rescued Purkinje neuronal death via activation of DOR (Lim et al., 2004). Zhao et al. (2006) also showed that pretreatment with DOR agonist Tan-67 reduced neuronal death in rat hippocampal slices exposed to OGD. In another study, preconditioning with DADLE before OGD treatment increased cortical neuronal survival via activating DOR, while naltrindole completely blocked the protective effect induced by DADLE (Ke et al., 2009). Zheng et al. (2012) reported that DADLE reduced the release of lactate dehydrogenase (LDH) and neuronal apoptosis in a concentration-dependent manner. Similarly, oxycodone, a DOR agonist, alleviated OGD-induced neuronal damage and increased neuronal viability in rat hippocampal neuron culture (Kong et al., 2019). Moreover, we found that DOR activation reduced OGD-injured ionic disruption in cortical neurons, e.g., increased K^+^ efflux and Na^+^, Ca^2+^ influx (Chao et al., 2007a, b, 2008).

Likewise, DOR plays a protective role in OGD-induced damage in astrocytes. DOR antagonist partially abolished the effect of DADLE and decreased cell viability (Wang et al., 2019).

#### Delta-opioid receptor neuroprotection against excitotoxic stress

In the *in vitro* neocortical model of glutamate-induced excitotoxic stress, we found that DOR activation with DADLE remarkably reduced neuronal death from glutamate-induced injury. Applying naltrindole simultaneously with DADLE in the cultures, the beneficial effect of DADLE was abolished (Zhang et al., 2000). In contrast, activating and inhibiting MOR or KOR in the same experimental conditions did not result in the same effect, implying that DOR, but not MOR or KOR, provided a unique protection against glutamate-induced neuronal injury (Zhang et al., 2000).

HPC increases the resistance of neurons to severe cytotoxic stress and makes them more tolerant. Rapid HPC was found to decrease glutamate-induced neurotoxicity, but this beneficial effect was reversed by the DOR antagonist naltrindole (Zhang et al., 2006). Rapid HPC increased DOR protein expression without altering DOR mRNA levels, suggesting the role of DOR in rapid HPC-induced neuroprotection against glutamate-induced injury (Zhang et al., 2006).

### Delta-opioid receptor protection in rat models

Several lines of experimental evidence suggest that DOR activity can ameliorate ischemia-induced brain damage in rats. Jeong et al. (2012) demonstrated that remifentanil activation of DOR reduced the infarct volume after MCAO. In 2002, Zhao et al. reported that electroacupuncture (EA) reduced ischemic brain injury via DOR. Later studies also validated that EA increased brain tolerance to ischemic damage via DOR (Xiong et al., 2007; Zhou et al., 2013). EA application reduced brain infarct volume and improved neurological outcomes after MCAO. Consistent with these findings, naltrindole treatment negated EA-mediated ischemic protection (Xiong et al., 2007; Zhou et al., 2013; Chen et al., 2014a). Moreover, the increased release of endogenous enkephalin in the striatum after EA stimulation, as noted after immunohistochemistry, may act on DOR to reduce ischemic injury (Xiong et al., 2007).

In the forebrain ischemia rat model, Su et al. (2007) revealed that DOR activation with DADLE, before forebrain ischemia, decreased hippocampal CA1 neuronal damage. Another study showed that DADLE treatment increased the number of survival neurons in hippocampal CA1 after forebrain ischemia (Wang et al., 2011). Some studies suggest that DOR protection in the context of forebrain ischemia may differ depending on the doses of DADLE, brain regions, and ischemic severity. Iwata et al. (2007b) reported that intraperitoneal administration of DADLE has limited neuroprotective efficacy against severe (10 minutes) forebrain ischemia in the hippocampus. Subsequently, they observed that pre-ischemia treatment with DADLE protected ischemia-resistant regions of CA3 and dentate gyrus, but the non-ischemia-sensitive region, CA1, during moderate (8 minutes) and mild (6 minutes) forebrain ischemia (Iwata et al., 2007a).

Likewise, DOR also has a protective effect in a rat model of global cerebral ischemia. Charron et al. (2008) showed that DOR stimulation by DPDPE increased the number of viable hippocampal CA1 neurons. DOR protein expression decreased in the global cerebral ischemic rat model caused by asphyxial cardiac arrest. Meanwhile, DADLE treatment reversed the down-regulation of DOR expression and attenuated hippocampal CA1 neuronal damage (Gao et al., 2010).

In addition, HPC has a protective effect on cardiac arrest (CA)-induced global ischemic injury (Gao et al., 2012). HPC induced prolonged neuroprotection, including improved neurological outcome and reduced neuronal loss following CA, while naltrindole abolished these protective effects. Moreover, HPC up-regulated neuronal DOR expression (Gao et al., 2012), further suggesting that DOR is involved in HPC-mediated neuroprotection against CA-induced brain injury.

The regulation of astrocyte contributed to the neuroprotection of DOR activation against ischemic brain injury (Duan et al., 2011). Activation of DOR by DADLE was found to enhance positive astrocyte response in the hippocampus of rats with global ischemia, leading to hypertrophy and increased ramification of processes. Additionally, DOR activation induced astroglial apoptosis, ultimately reducing the number of dying astrocytes and promoting neuronal survival (Duan et al., 2011).

DOR exhibits neuroprotective effects on motor function impairment in spinal cord ischemia. Pre-treatment with the DOR agonist, SNC80, enhanced hind-limb motor function outcomes in rats (Horiuchi et al., 2004). Turner and Johnson (2011) revealed that DOR activation before, during, and after spinal OGD prolonged respiratory motor output in the neonatal rat.

### Delta-opioid receptor protection in mouse models

DOR has a protective effect on neurological damage caused by hypoxic and ischemic spinal cord injury in mouse models.

#### Delta-opioid receptor neuroprotection against hypoxic injury

Some early studies suggested that DOR agonists induced a neuroprotection against hypoxic injury in mice. For example, DOR activation with DPDPE enhanced the survival time of mice subjected to hypoxic treatment (Mayfield and D’Alecy, 1994). Consistent with this observation, Bofetiado et al. (1996) showed that DOR activation with BW373U86 increased the survival time of mice during hypoxic injury. However, Mayfield et al. (1996) found that the level of DOR decreased after prolonged hypoxia for 7 days. Moreover, there were other studies that reported opposite results. *In vivo* studies (Hosobuchi et al., 1982; Skarphedinsson and Thorén, 1988) showed that opioid antagonists, such as naloxone that binds non-specifically to DOR, MOR and KOR, induced neuroprotection, implying that opioid activation caused neuronal damage. We resolved the controversy in 1999–2000 by improved experimental approaches and demonstrated the role of DOR in neuroprotection (Zhang et al., 2000; Chao and Xia, 2010; He et al., 2013).

DOR and γ-aminobutyric acid (GABA) receptors have neuroprotective effects on the mature cortex (Zhao et al., 2005; Chao et al., 2007a; Tian et al., 2013). We found that DOR over-expression led to a decrease in GABA receptor expression and prevented the hypoxia-induced increase in GABA receptor expression in the mouse cortex, suggesting that DOR and GABA achieved neuroprotection against hypoxic injury by maintaining mutual balance (Feng et al., 2011).

#### Delta-opioid receptor neuroprotection against ischemic injury

DOR activation is beneficial for ischemia-induced brain damage in mice. Pretreatment with DOR agonist Delt-D(var) decreased cerebral infarct size and improved behavioral outcomes in mice following MCAO-induced ischemia (Govindaswami et al., 2008). Likewise, administration of Tan-67 not only reduced infarct volume and neuronal loss, but also enhanced mice survival and behavioral recovery after MCAO in cerebral ischemic mice (Min et al., 2018).

#### Delta-opioid receptor neuroprotection against spinal cord injury

DOR activation with DADLE improved motor functional recovery in a mouse model of spinal cord injury. Chen et al. (2023) found that DADLE-treated mice had smaller lesions, higher BMS scores, and better footprint analysis results compared to the injury group, reversing the increased toe-dragging ratio.

### Delta-opioid receptor protection in other animal studies

DOR activation has been shown to have neuroprotective properties in hamsters and rabbits. In hibernating hamsters, DOR activation helps neuronal survival and maintains CNS function. DPDPE, a hibernation-regulating substance, activates DOR and reduces hippocampal neuron death in hamsters exposed to low temperatures (Tamura et al., 2006). In rabbits with spinal cord ischemic injury, administration of DADLE reduces neuron loss and improves behavioral performance (Liu et al., 2015).

### Delta-opioid receptor protection against neurodegenerative injury

A series of literature reports that DOR and its agonists contributed to the protection of neurodegenerative diseases, such as PD and AD.

#### Alzheimer’s disease

The protective effect of DOR on AD is still being studied. Mathieu-Kia et al. (2001) found a decrease in DOR expression in the amygdaloid complex and ventral putamen of postmortem brains in AD patients, suggesting a potential role of DOR in AD pathophysiology. Our study suggests that DOR has a protective effect against AD-related damage, as DOR inhibition worsens PC12 cell damage induced by Aβ_1–42_ oligomers (Xu et al., 2020a).

Recently, we found in an AD mouse model that DOR activation decreased the deposition of Aβ plaques in the cortex and hippocampus, and reduced neuroinflammation, thus inhibiting neuronal apoptosis and improving cognitive impairment (Xu et al., 2025).

#### Parkinson’s disease

Mounting evidence suggests that DOR has anti-parkinsonism effects (Hille et al., 2001; Hallett and Brotchie, 2007; Begum and Sen, 2018). Rats treated with dopamine D2 or D1 antagonists, followed by activation of DOR with SNC80, experienced a complete reversal of dopaminergic antagonist-induced catalepsy. SNC80 also reversed akinesia in rats treated with reserpine (Hille et al., 2001). Additionally, DOR binding density showed a significant increase in the premotor cortex, sensorimotor striatum, and associative striatum in the reserpine-induced PD rat model, implying that DOR may be a compensatory mechanism for the dopamine depletion resulting from reserpine treatment (Hallett and Brotchie, 2007).

DOR activation with SNC80 in MPTP-treated marmoset increased motor activity and alleviated Parkinsonian symptoms (Hille et al., 2001). Additionally, SNC-80 improved tremors and motor impairments in the MPTP-induced PD mice model (Begum and Sen, 2018).

However, different doses and brain regions of DOR agonist injection manifested different effects on the treatment of PD (Mabrouk et al., 2009). UFP-512 has dual regulation of motor activity in hemiparkinsonian rats. UFP-512 enhanced motor action at low doses but induced motor inhibition at higher doses. Additionally, the administration of UFP-512 in the globus pallidus resulted in increased akinesia, while weakening akinesia in the substantia nigra reticulate (Mabrouk et al., 2009).

*In vitro* cell experiments further confirmed the protective effect of DOR on PD. We found that DOR activation increased cell viability and decreased LDH release in HEK293T cells under hypoxia or MPP^+^ injury (Chen et al., 2014b). 

## Delta-Opioid Receptor and Neural Regeneration

Neural regeneration plays an important role in repairing damage to nerve function. As is well known, brain-derived neurotrophic factor (BDNF) plays an important role in regulating synapse plasticity (Camuso et al., 2022). DOR activation improved synaptic morphology and also regulated BDNF to promote neuronal regeneration. Zhang et al. (2021a) found that cerebral ischemia can lead to the destruction of synaptic structures, thereby damaging synaptic transmission. DOR activation with DADLE increased the density of dendritic spines and improved synaptic transmission in I/R injury depending on BDNF signaling.

**Figure 1:**
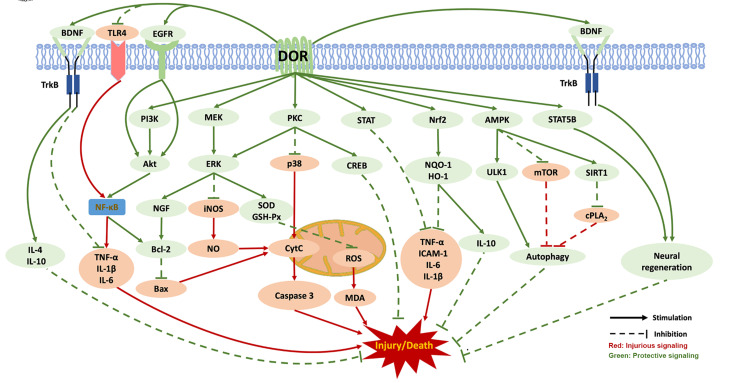
The molecular mechanisms of DOR-mediated neural protection and regeneration. The green boxes represent the neuroprotective or regenerative signaling after DOR activation, while the orange boxes represent the factors that exert cell damage under different stresses. The blue box represents the bidirectional action of NF-κB, depending on the types of injury and/or cells. When cells are subjected to different stress stimuli, the expression of injurious molecules is upregulated (orange boxes), while DOR activation increases the expression of protective or regenerative molecules as a protective or regenerative strategy (green boxes). Arrows indicate the increased expression/activity of the downstream molecules and dashed lines represent an inhibitory effect on the downstream molecules. The red arrow/dish lines show the injury caused by stress, such as ischemia and hypoxia, while the green arrow/dish lines indicate the protective or regenerative signaling induced by DOR. Akt: Protein kinase B; AMPK: AMP-activated protein kinase; Bcl-2: B cell lymphoma 2; BDNF: brain-derived neurotrophic factor; cPLA2: cytosolic phospholipase A2; CREB: cyclic AMP-response element binding protein; CytC: cytochrome c; DOR: delta-opioid receptor; EGFR: epidermal growth factor receptor; ERK: extracellular signal-regulated protein kinases; GSH-Px: glutathione peroxidase; HO-1: heme oxygenase 1; ICAM-1: intercellular adhesion molecules-1; IL: interleukin; iNOS: inducible nitric oxide synthase; MDA: malondialdehyde; NF-κB: nuclear transcription factor-kappa B; NGF: nerve growth factor; NO: nitric oxide; NQO-1: NADPH quinone oxidoreductase-1; Nrf2: nuclear factor erythroid 2-related factor 2; PI3K: phosphatidylinositol 3-kinase; PKC: protein kinase C; ROS: reactive oxygen species; SIRT1: sirtuin 1; SOD: superoxide dismutase; STATs: signal transducer and activator of transcriptions; TrkB: tropomyosin-related kinase B; TNF-α: tumor necrosis factor α; TLR4: Toll-like receptor 4.

Neural differentiation is a key process in neural regeneration (Chuma et al., 2024). Narita et al. (2006) found that in multipotent neural stem cells derived from the forebrain of embryonic C3H mice, DOR agonist SNC80 significantly enhanced neural differentiation from neural stem cells into MAP2ab-positive neurons via BDNF/ tropomyosin-related kinase B (TrkB) pathway, while the MOR agonist DAMGO and the KOR agonist U50, 488H did not show similar effects. This result suggests that DOR may play a unique role in regulating neural differentiation mechanisms (**Figure 1**).

Accumulating evidence suggests that the signal transducer and activator of transcription (STAT) pathway regulates neuronal differentiation (Wu et al., 2022; Qian et al., 2024). Georganta et al. (2013) demonstrated that DOR agonists enhanced cell survival, promoted neurite outgrowth, and facilitated neuronal differentiation, resulting in elevated expression of neuronal marker β-III tubulin in Neuro-2A cells. Naltrindole or transfecting the cell with a dominant-negative mutant of the STAT5B gene (DN-STAT5B) blocked this cytoprotective aspect of DOR. The results suggest that the STAT5B-dependent pathway is involved in DOR-mediated neurotropic events (Georganta et al., 2013; **Figure 1**).

DOR enhances neuroprotection by improving neurogenesis and aiding in the recovery of spatial cognitive function following global ischemic injury (Wang et al., 2016). DADLE was found to improve spatial learning and memory in rats, as well as promote the proliferation and differentiation of newly formed neurons in the CA1 and dentate gyrus regions of global ischemia rats. Naltrindole was shown to block this effect, suggesting that DOR-mediated increase in neurogenesis may play a role in improving neuronal tissue post-ischemic injury (Wang et al., 2016).

## Mechanisms for Delta-Opioid Receptor Neuroprotection

The neuroprotective phenomenon of DOR is gaining more recognition, and researchers are extensively investigating the underlying mechanisms. Current literature divides DOR protection mechanisms into the maintenance of ion homeostasis, inhibition of intracellular death signals, activation of protective pathways, regulation of intracellular proteins and molecules, enhancement of antioxidant function, and inhibition of inflammatory signaling pathways (**Figure 1**).

### Delta-opioid receptor stabilization of ionic homeostasis across the cellular membrane

The maintenance of adept neuronal functioning relies on ionic homeostasis (Rasmussen et al., 2020). Acute hypoxic–ischemic (HI) stress may lead to the disruption of ionic balance, resulting in increased K^+^ efflux and influx of Na^+^ and Ca^2+^, ultimately leading to neuronal damage and death (Chao and Xia, 2010). The imbalance of ion homeostasis caused by abnormal changes of K^+^ is an initial and key change of HI-induced neuronal damage (Chao and Xia, 2010). Our laboratory first clarified that DOR activation attenuated the increase of extracellular K^+^-caused neuron death. The addition of a DOR antagonist reversed the protection of DOR activation on K^+^ homeostasis (Chao et al., 2007b). However, MOR activation was ineffective in hypoxia-induced K^+^ derangement, suggesting that DOR, but not MOR plays a critical role in maintaining K^+^ membrane homeostasis under hypoxia (Chao et al., 2007a).

Na^+^ is a crucial effector in neuronal depolarization and conduction of action potential that is essential for maintaining membrane potential (Wilbers et al., 2023). The substantial influx of Na^+^ caused by hypoxia could indirectly stimulate K^+^ leakage (Chao and Xia, 2010). The major mechanism of DOR attenuating K^+^ derangement is related to the inhibition of Na^+^ influx in the cortex. The mechanism of DOR-mediated neuroprotection against K^+^ disruption was blocked by low-Na^+^ perfusion, either with impermeable N-methyl-D-glucamine or permeable Li^+^ substitution (Chao et al., 2008). Moreover, we found that in the regulation of Na^+^ channels, DOR activation played an inhibitory role, as reflected by the prohibition of inward Na^+^ currents in DOR and Na1.2 channels co-expressed *Xenopus* oocytes after DOR activation (Kang et al., 2009). Hence, we thought that DOR-induced protection against the hypoxic K^+^ disruption is partially attributed to Na^+^ channel regulation. In additional studies, we found that DOR activation reduced anoxic K^+^ leakage by inhibiting Na^+^ influx mediated through Na^+^ voltage-gated channels. Na^+^ channel inhibitor TTX is responsible for blocking this effect (Kang et al., 2009; Chao et al., 2012).

Additionally, our data showed that DOR activation suppressed the hypoxia-induced increase in Ca^2+^ entry-BK channel activities, thereby helping to alleviate the disruption of K^+^ homeostasis caused by hypoxia (Chao et al., 2007a). Our result revealed that the absence of extracellular Ca^2+^ attenuated hypoxic K^+^ disruption (Chao et al., 2007a). Inhibition of BK channels and KATP channels attenuated K^+^ disorder in the cortex. However, in the absence of calcium perfusion, DADLE did not further reduce the anoxia-induced disruption of K^+^, indicating that the DOR effect is partially mediated through the regulation of Ca^2+^ entry (Chao et al., 2007a). Furthermore, DOR activation did not further attenuate K^+^ derangement induced by anoxia with the BK channel blocker, paxilline. However, the inhibition of KATP channels has no influence on DOR protection from K^+^ disruption under anoxia (Chao et al., 2007a). These results demonstrated that DOR activation protects K^+^ homeostasis via regulating Ca^2+^ entry-BK channel activity.

In addition, DOR activation inhibited the ionic reactions via targeting ionotropic glutamate NMDA receptors (Pamenter and Buck, 2008). DOR activation inhibited Na^+^ entry mediated by NMDA receptors, leading to the stabilization of K^+^ levels. This effect is abolished by NMDA receptor inhibitor MK801. DOR antagonist with naltrindole potentiated NDMA receptor activity and increased intracellular Ca^2+^ during anoxia in turtle cortical neurons, suggesting that DOR inhibited NMDA receptor-mediated Ca^2+^ influx under anoxic injury (Pamenter and Buck, 2008). These findings suggest that the stabilization of DOR ionic homeostasis is essential for executing neuroprotection in cases of HI injury.

### Delta-opioid receptor’s enhancement of survival signaling

Intricate survival mechanisms have been proposed to explain the neuroprotection of DOR activation, which includes activation of protein kinase C (PKC)/extracellular signal-regulated protein kinases (ERK), phosphatidylinositol 3-kinase (PI3K)/protein kinase B (Akt) pathways, and the transactivation pathway of epidermal growth factor receptor (EGFR), as well as the regulation of transcription factors and trophic factors.

#### Mitogen-activated protein kinase signaling

The mitogen-activated protein kinase (MAPK) family has three members: ERK, c-Jun-N-terminal kinase, and p38 MAPK (Zhang et al., 2020). Cumulative evidence suggests that DOR activation induces an increase in phosphorylated ERK levels, which is implicated in the DOR-mediated protection against hypoxic/ischemic injury (Ma et al., 2005; Hong et al., 2007). In serum-deprived PC12 cells and ischemic rat brain slices, a low concentration of DADLE promoted cell survival. Administration of PD98059 post-treatment, an inhibitor of ERK kinase (MEK), blocked the protective effect of DADLE and ERK phosphorylation (Hayashi et al., 2002; Zheng et al., 2012). DADLE-induced reduction in LDH leakage during OGD-induced neuronal injury was blocked by the ERK inhibitor, U0126, indicating that DOR promoted neuronal survival by activating ERK signaling (Ke et al., 2009). Similarly, DOR activation with oxycodone attenuated OGD-induced hippocampal neuronal injury via enhancing ERK1/2 phosphorylation, which is then blocked by naltrindole (Kong et al., 2019).

Moreover, our study showed that DOR activation led to an elevation in excitatory amino acid transporters (EAATs) through the MAPK pathway (Liang et al., 2014). Administration of UFP-512 up-regulated the mRNA and protein expression of EAAT1 and EAAT2, but not EAAT3, and thus accelerated astrocytic glutamate uptake. More importantly, inhibitors of DOR, MEK, ERK, and p38 reversed DOR-induced increases in EAAT1 and EAAT2 (Liang et al., 2014). DOR could potentially defend neurons by enhancing astrocytic EAATs via the MEK-ERK-p38 pathway.

#### Protein kinase C signaling

PKC activity is the downstream signal of DOR-mediated G protein function (Zhang et al., 1999). DOR neuroprotection against hypoxic injury in neurons requires the PKC-dependent pathway (Ma et al., 2005). Our laboratory found that HPC treatment elevated pERK expression in severe hypoxia, while PKC inhibitors blocked this result. Indeed, DOR-mediated HPC neuroprotection depends on PKC/ERK cascades (Ma et al., 2005). Zhu et al. (2011) reported that DOR activation with DADLE improved NaN_3_-induced mitochondrial dysfunction in rat cortical neurons through the ERK pathway, specifically reducing mitochondrial membrane depolarization, decreasing mitochondrial Ca^2+^ overload and reactive oxygen species (ROS) generation, and inhibiting cytochrome c (CytC) release and caspase 3 activation. Moreover, PKC, but not PKA inhibitors abolished DADLE-induced elevation of ERK levels, suggesting the crucial involvement of the PKC/ERK pathway in NaN_3_-induced mitochondrial dysfunction in rat cortical neurons (Zhu et al., 2011).

The PKC pathway is also involved in DOR-mediated stability of ionic homeostasis. Chao et al. (2007b) found that DOR-attenuated K^+^ efflux depends on the PKC pathway, while the PKA-independent pathway in mouse cortical slices responds to anoxia and OGD. In agreement with this observation, DOR inhibited Na^+^ influx induced by HI injury via the PKC pathway, especially PKCβII and PKCθ isoforms (Chao et al., 2012).

#### Phosphatidylinositol 3-kinase/protein kinase B signaling

PI3K, as a downstream signal of DOR, is required for Akt activation in neurons or neuron-like cells. In *in vitro* studies, DOR activation with N-desmethylclozapine increased cell survival and reduced apoptosis in the NG108-15 cell line via the PI3K/Akt pathway (Olianas et al., 2011). Application of PI3K and Akt inhibitors to PC12 cells reduced the DADLE-mediated increase in neurite length, number, and cell survival (Sen et al., 2013). In primary cultured rat cortical neurons treated with NaN_3_ and subjected to prolonged DADLE stimulation, DALDE was found to reduce cell injury and provide neuroprotection by activating the PI3K/Akt/nuclear transcription factor-kappa B (NF-κB) pathway (Zhu et al., 2018).

In an *in vivo* study, DOR activation with SNC-121 protected retinal ganglion cells (RGC) against glaucomatous injury depending on the PI3K/Akt pathway in a rat model of chronic glaucoma (Husain et al., 2017).

#### Epidermal growth factor receptor transactivation pathway

Evidence has suggested that DOR leads to EGFR transactivation and induces neuroprotection, which differs from the classical Gαi/o-dependent pathway of DOR (Zhang et al., 2015). EGFR can mediate downstream signals of DOR activation through the metalloproteinase (MMP)-dependent EGF-like growth factor (HB-EGF) excretion pathway, also known as transactivation. An *in vitro* study revealed that DOR activation contribute to MMP-dependent cleavage of HB-EGF from the membrane, leading to its interaction with EGFR for downstream pathway activation (Cohen et al., 2007). Chen et al. (2021a) showed that DADLE improved the neurologic deficits and reduced infarct volume induced by ischemic injury. The effects were prevented by EGFR inhibitor and MMP inhibitor. Moreover, DADLE increased EGFR and HB-EGF release, as well as downstream signaling through ERK1/2 and Akt expression. These effects were blocked by EGFR and MPP inhibitors, suggesting that DOR exerts neuroprotection through MMP-mediated HB-EGF secretion followed by EGFR activation (Chen et al., 2021a).

#### Regulation of transcription factors

DOR activation mediates cell biological events involving nuclear factor erythroid 2-related factor 2 (Nrf2) and cyclic AMP-response element binding protein (CREB).

*Nuclear factor erythroid 2-related factor 2 pathway*

Nrf2 plays an important role in antioxidation and anti-inflammation (Kim, 2021; Wang and He, 2022). Our research revealed that DOR activation with UFP-512 led to increased translocation of Nrf2 from the cytoplasm to the nucleus, resulting in the up-regulation of Nrf2-regulated genes, such as NADPH quinone oxidoreductase-1 (NQO-1), heme oxygenase 1 (HO-1), and glutamate-cysteine ligase modifier. The effect of DOR on Nrf2 nuclear translocation was reversed by naltrindole (Cao et al., 2015).

*Cyclic AMP-response element binding protein pathway*

CREB is a universal transcription factor regulating neuronal growth, differentiation, and survival (Sharma and Singh, 2020). DOR activation increased CREB phosphorylation in NG108-15 cells (Bilecki et al., 2000). The phosphorylation of CREB was entirely blocked by DOR antagonist naltrindole and PKC inhibitor, suggesting that stimulation of DOR induced CREB phosphorylation involving the PKC pathway (Bilecki et al., 2000). In rat cerebral ischemic injury, Yang et al. (2014) discovered that pCREB expression increased in the hippocampus after ischemia. DOR activation with BW373U86 further elevated pCREB expression and decreased neuronal injury, an effect abolished by naltrindole. Our data demonstrated that DOR activation with UFP-512 enhanced the expression of pCREB and attenuated α-synuclein overexpression and its oligomer formation with cell injury induced by MPP^+^/hypoxia in HEK 293T cells (Chen et al., 2014b). These findings support that transcription factor CREB is a potential mechanism of DOR-mediated neuroprotection under stress.

#### Regulation of trophic factors

Neurotrophic factors are essential for maintaining the survival and growth of neurons, as well as for promoting the differentiation of neurons and synapses (Severini, 2022). They are mainly composed of BDNF and nerve growth factor (NGF). DOR has been shown to protect neurons by up-regulating neurotrophic factor expression.

*Brain-derived neurotrophic factor pathway*

BDNF pathway is a vital neuroprotective regulator that increases neuronal tolerance subjected to hypoxia/ischemia (Xiong et al., 2021). DOR activation increased the expression of mRNA BDNF in the cortex, while naltrindole removed this increase in BDNF mRNA (Torregrossa et al., 2004). Similarly, Chomanic et al. (2022) showed that DOR activation with endogenous opioid peptides stimulated BDNF expression in the hippocampus.

*Nerve growth factor pathway*

NGF could induce the expression of DOR in PC12 cells (Abood and Tao, 1995). Moreover, treatment with DADLE up-regulated NGF expression in the hippocampus, midbrain, PC12 cells, and F11 cells of mice (Hayashi and Su, 2003). Sen et al. (2013) confirmed that there is crosstalk between DOR and NGF in neuroprotection and differentiation. The DOR mRNA level and cell survival of PC12 cells and F11 cells were remarkably higher in the presence of NGF than in the absence of NGF, suggesting that DOR-mediated neuroprotection is related to NGF expression. Moreover, PI3K inhibitor LY and MAPK inhibitor PD reversed the DADLE effect. These results describe that MAPK and PI3K/Akt pathways are involved in the function of NGF-regulated DOR-mediated neuroprotection (Sen et al., 2013). Moghal et al. (2018) also confirmed that DADLE promoted neuronal survival via up-regulating MEK-ERK1/2-NGF-Bcl2 pathway in SH-SY5Y cells subjected to endoplasmic reticulum (ER) stress.

*Regulation of B-cell lymphoma 2*

B-cell lymphoma 2 (Bcl-2), a key regulator in the anti-apoptosis pathway, plays a crucial role in cell survival regulation (Li et al., 2020). In severe hypoxia-treated neurons, we found that treatment with HPC elevated the level of Bcl-2 in neurons and subsequently prevented the release of cytochrome c from mitochondria. Naltrindole blocked this effect, suggesting that DOR-mediated HPC neuroprotection is associated with Bcl-2 (Ma et al., 2005).

Under ischemic injury, the activation of DOR by oxycodone significantly increased the expression of Bcl-2 in hippocampal neurons exposed to OGD (Kong et al., 2019). Wang et al. (2019) also showed that DOR activation inhibited apoptosis by increasing the protein expression of Bcl-2 in astrocytes induced by OGD.

Moreover, prolonged DADLE stimulation increased the levels of histone acetylation on the Bcl-2 promoter, leading to enhanced Bcl-2 expression in NaN_3_-treated neurons (Zhu et al., 2018).

### Delta-opioid receptor-mediated inhibition of apoptotic/death/necroptosis/ferroptosis signaling

DOR can activate intracellular survival signaling and also induce endogenous apoptosis, death, necroptosis, and ferroptosis signaling, exerting protective effects by inhibiting p38 MAPK signaling and apoptotic molecules.

#### p38 MAPK pathway

p38 MAPK is a key intracellular transduction pathway regulating cellular stresses and apoptosis. Substantial data suggest that DOR activation exerts neuroprotection by restraining the p38 MAPK pathway. DOR regulated HPC-mediated neuroprotection by inhibiting the PKC-p38-cytochrome c pathway and counteracting ERK activation in neurons to protect against severe hypoxic injury. The crosstalk between ERK and p38 exhibits a “*Yin-Yang*” antagonism under the regulation of DOR-G protein-PKC pathway (Ma et al., 2005). Anisomycin at 50 ng/mL inhibited the increase of p38 immunoreactivity in cortical neurons exposed to hypoxic and naltrindole insults, reconfirming that DOR is a promoter of anoxic damage (Hong et al., 2007). In cortical neurons exposed to OGD, DADLE inhibited the up-regulation of p38 levels caused by ischemic injury. The reversal of this effect by naltrindole suggests that DOR protects against neuronal injury by blocking the p38 MAPK pathway (Ke et al., 2009). The chronic ocular hypertensive rat model showed that the DOR agonist SNC-121 could protect RGC function against glaucomatous injury by reducing p38 MAPK activity, thus exerting retina neuroprotection (Abdul et al., 2013).

#### Apoptosis pathways

The caspase family is crucial for inducing apoptosis/death (Vitale et al., 2023). Various data suggest that DOR activation has the potential to influence the caspase pathway and mitigate neuronal cell death. Applying DADLE promoted cell survival against OD injury in cortical neurons and glial cells via inhibiting caspase-3/7 activity (Kim et al., 2012). UFP-512 protected PC12 cells from hypoxia or MPP^+^ damage by prohibiting the production of cleaved caspase-3 (Xu et al., 2019). Guney et al. (2019) showed that DOR-mediated HPC induced neuroprotection in cortical neurons against severe hypoxia by reducing caspase 3 immunoreactivity. Caspase 3 is also a vital indicator of ischemic injury. DADLE inhibited activation of caspase 3 in MCAO-induced ischemic injury in rats (Yang et al., 2009). Furthermore, the activation of DOR using SNC80 inhibited the rise of cleaved caspase 3 in neural cells undergoing H2O2-induced apoptosis (Narita et al., 2006).

DADLE prevented the translocation of Bax (a pro-apoptotic factor) into mitochondrion and its oligomerization induced by MTPT injury, indicating that DADLE offered neuroprotection by inhibiting the Bax-related apoptotic pathway in brain tissue of CD-1 mice (Tsao and Su, 2001). DADLE also induced autophagy by inhibiting apoptosis signaling via the reduction of Bax protein expression in rat astrocytes induced by OGD (Wang et al., 2019). Pretreatment with DOR agonist oxycodone reduced Bax production caused by OGD-treated hippocampal neurons (Kong et al., 2019).

#### Necroptosis pathway

Necroptosis, a form of cell death, can exacerbate cell death and neuroinflammation in the nervous system. However, DOR activation has a neuroprotective effect against necroptosis after spinal cord injury. Treatment with DADLE resulted in a decrease in the protein levels of necroptosis markers, including RIPK1, RIPK3, and MLKL, leading to enhanced recovery of motor function (Chen et al., 2023).

#### Ferroptosis pathway

Ferroptosis is a new type of programmed cell death that is triggered by iron-dependent phospholipid peroxidation (Liang et al., 2022). Cai et al. (2023) found that DOR signaling inhibited ferroptosis, which depends on the Nrf2 pathway in MPTP-induced PD models. DADLE increased the levels of key proteins GPX4 and SLC7a11 that regulate ferroptosis, and reduced the accumulation of lipid peroxidation contents malondialdehyde and 4-HNE.

### Delta-opioid receptor-mediated regulation of oxidative stress

Hypoxic/ischemic injury induces the generation of nitric oxide (NO) and ROS, leading to oxidative damage (He et al., 2013; Chen et al., 2022b). The activation of DOR was shown to mitigate oxidative injury in the rat brain following ischemic damage by increasing the activity of antioxidant enzymes (e.g., superoxide dismutase and glutathione peroxidase) and by reducing the production of free radicals such as malondialdehyde and NO (Yang et al., 2009). DOR agonist DPDPE and SNC80 decreased the dichlorofluorescein-mediated fluorescence, which is the marker for oxidative stress caused by intracellular free radical generator SIN-1 or the HIV-related protein TAT in SK-N-SH cells (Wallace et al., 2006). Additionally, in the rat model of glaucomatous injury, DOR activation with SNC-121 resulted in the protection of RGCs by reducing NO production through the inhibition of inducible nitric oxide synthase (Husain et al., 2014). Activation of DOR is crucial in attenuating MPTP-induced oxidative stress. SNC-80 decreased protein carbonyl content and enhanced mitochondrial complex-I activity through the Nrf-2/HO-1 antioxidant pathway (Begum and Sen, 2018).

Naltrexone, by inhibiting MOR, reduced oxidative markers 3-nitrotyrosine and inducible nitric oxide synthase, leading to a neuroprotective effect following traumatic brain injury (Rodriguez et al., 2024).

### Delta-opioid receptor-mediated immunomodulatory mechanism

The immune response serves as a protective process against pathogens and tissue damage (Chi et al., 2024). However, excessive immune responses such as inflammation have adverse effects on the structure and function of neurons (Woo et al., 2024). The neuroinflammatory process is seen as a crucial event in the hypoxia and ischemia, chronic glaucoma model, and neurodegenerative disease (Yang et al., 2020; Chen et al., 2022a; Ohm et al., 2024). DOR, as an immunomodulator, reduces the nerve damage caused by neuroinflammation by regulating the secretion of inflammatory factors and the inflammatory signal pathway (Wang et al., 2014; Begum and Sen, 2018; Qiu et al., 2019; Geng et al., 2020; Fu et al., 2021; Husain et al., 2021).

#### Inhibition of pro-inflammatory cytokines

HI can trigger an inflammatory response, aggravating the cerebral injury. DOR is a potential target to regulate inflammatory cytokines to attenuate HI damage. In the *in vitro* experiment, hypoxia resulted in different changes in tumor necrosis factor α (TNF-α) levels in glial and PC12 cells. Specifically, TNF-α increased in astrocytes but decreased in PC12 cell. The hypoxic injury in PC12 cells was exacerbated by the exogenous addition of TNF-α. More importantly, DOR activation reversed the hypoxia-induced changes in TNF-α expression in both cells. Therefore, DOR inhibited the inflammatory response induced by TNF-α with varying reactions in astrocytes and PC12 cells under severe hypoxia (Wang et al., 2014).

The level of TNF-α remarkably increased after ischemic injury. DOR activation with remifentanil reduced the upregulation of TNF-α expression caused by cerebral ischemic injury in rats and this DOR effect was abolished by naltrindole (Jeong et al., 2012). In glaucomatous injury, DOR activation with SNC-121 achieved neuroprotection by inhibiting TNF-α generation (Abdul et al., 2013).

#### Regulation of inflammatory signaling

*Nuclear factor erythroid 2-related factor 2 pathway*

One of the most important functions of transcription factor Nrf-2 is to suppress inflammatory reactions. We reported that HI induced an upregulation of the Nrf-2 nuclear protein and its downstream anti-inflammatory genes, including HO-1 and NQO-1 (Qiu et al., 2019). DOR activation also elevated the level of Nrf-2, HO-1, and NQO-1. The expression of pro-inflammatory cytokines such as TNF-α and IL-6, along with intercellular adhesion molecules-1, was decreased by DOR signaling. Conversely, the level of the anti-inflammatory factor IL-10 was increased. This suggests that DOR-mediated neuroprotection triggers an anti-inflammatory response through the activation of the Nrf-2/HO-1/NQO-1 signaling pathway (Qiu et al., 2019).

*Toll-like receptor 4 pathway*

Toll-like receptor 4 (TLR4) plays an important role in innate immunity and participates in the inflammatory response. NF-κB, a downstream molecule activated by TLR4, can regulate the expression of inflammatory cytokines. It has been reported that TLR4 is involved in the pathological process of ischemic injury. Administration of DADLE inhibited the increase in TLR4 and NF-κB levels following cerebral ischemic injury in rats (Fu et al., 2021). DADLE also down-regulated the expression of pro-inflammatory cytokines TNF-α and IL-6 (Fu et al., 2021). Thus, DOR may ameliorate cerebral impairment by inhibiting the TLR4-mediated inflammatory mechanism.

*Brain-derived neurotrophic factor pathway*

The BDNF/TrkB pathway is involved in DOR-mediated anti-inflammatory response. DOR activation decreased TNF-α levels by up-regulating the BDNF/TrkB pathway in C57BL/6J mice cortex exposed to hypoxia (Tian et al., 2013). Geng et al. (2020) demonstrated that EA reduced the I/R-induced inflammatory response by activating the DOR-BDNF/TrkB pathway, resulting in a decrease in pro-inflammatory cytokines (TNF-α, IL-1β, and IL-6) and an increase in anti-inflammatory cytokines (IL-4 and IL-10) in PC12 cells following OGD impairment.

*Signal transducer and activator of transcription 3 pathway*

STAT3 is crucial for the inflammatory response mediated by DOR. Husain et al. (2021) reported that rats treated with SNC-121 reduced the production of pro-inflammatory cytokines (e.g., IL-1β and IL-6) in response to glaucomatous injury via inhibiting the phosphorylation of STAT3 at tyrosine 705 and increasing the acetylation of STAT3 at lysine 686.

### Delta-opioid receptor-induced regulation of microRNAs

MicroRNAs (miRNAs) play a crucial role in regulating cell survival and function (Zhang et al., 2021b). Increasing evidence suggests that the protective mechanism of DOR is related to modulating miRNAs under stress. Hypoxia and DOR can regulate 19 miRNAs in all miRNAs we examined. Of them, 17 miRNAs have different responses to hypoxia or DOR activation in the rat cortex, which target pathways influencing ion transport, axonal guidance, apoptosis, and other functions. For example, UFP-512 down-regulated the expression of miR-363 and miR-370, which are associated with neurexin signaling affecting ionic homeostasis (Yang et al., 2012). DOR activation with DADLE differentially regulated 93 miRNAs in SH-SY5Y cells exposed to the ER stress. Moghal et al. (2018) selected three candidate genes to verify the role of miRNA in DADLE regulation. For example, miR-200c is believed to promote cell survival under ER stress. Consistent with this, DADLE up-regulated miR-200c by 2.5–3 fold in SH-SY5Y cells following ER stress, and the cell viability was dampened by miR-200c inhibitor. Conversely, miR-30c-2-3p and miR-34b-5p have been shown to regulate unfolded protein response (*UPR*) genes. DADLE downregulated both miR-30c-2-3p and miR-34b-5p significantly during ER stress. However, the overexpression of miR-30c-2-3p mimics significantly inhibited the DADLE-induced increase in cell viability. In contrast, the overexpression of miR-34b-5p had no effect on this process, indicating their differential roles in DOR-mediated cellular events (Moghal et al., 2018). Thanjeem Begum et al. (2019) found that several novel miRNAs, such as xxx-m0073-3p, xxx-m0225-3p, and xxx-m0088-3p, enhanced DOR-induced neuronal survival under ER stress conditions.

### Delta-opioid receptor modulation of autophagy

Autophagy is an intracellular metabolic process that endows cytoprotection against various pathological damages. DOR activation using DADLE has been proven to provide neuroprotective benefits by stimulating autophagy, resulting in an elevation of the LC3-II/LC3-I ratio and a decrease in p62 levels in neuronal cells exposed to OGD. Treatment with an autophagy inhibitor, 3-methyladenine, or naltrindole resulted in the blocking of the beneficial effects of DADLE (Wang et al., 2019; Lai et al., 2020).

AMP-activated protein kinase (AMPK)-mediated autophagy plays an important neuroprotective role in cerebral ischemic injury (Sun et al., 2020). Administration of DADLE increased the phosphorylation level of AMPK and its downstream molecule ULK1 and decreased the phosphorylation level of mTOR. The addition of an AMPK inhibitor, compound C, reversed the protective effect of DADLE against ischemia. These results suggested that DOR-mediated autophagic activity depended on the AMPK/mTOR/ULK1 signaling axis (Lai et al., 2020). Chen et al. (2023) demonstrated that DADLE improved autophagic flux dysfunction by restoring lysosomal function impairment after spinal cord injury. DADLE treatment decreased lysosomal membrane permeabilization and reduced the phosphorylation of cytosolic phospholipase A2. The AMPK pathway is activated by DADLE treatment, leading to an increase in AMPK and sirtuin 1, while reducing p38 phosphorylation. The protection of DADLE was reversed by blocking the AMPK-sirtuin 1-p38-cytosolic phospholipase A2 pathway. DOR activation can promote autophagic flux through the AMPK-dependent pathway (Chen et al., 2023).

### Delta-opioid receptor-mediated regulation of mitochondria

Mitochondria is a critical organelle for maintaining biological function. The disorder of mitochondrial function is involved in the pathogenesis of stroke and neurodegenerative diseases (Harrington et al., 2023). DOR activation plays an essential role in maintaining mitochondrial function. DOR activation provided neuroprotection via regulating mitochondrial K_ATP_ channel activation and mitochondrial electron transport system in Purkinje cells (Lim et al., 2004). Zhu et al. (2009) also demonstrated that DOR signaling ameliorated NaN_3_-induced cortical neuron damage by inhibiting mitochondrial respiratory chain injury. Moreover, the activation of DOR with enkephalin enhanced neuronal survival by enhancing mitochondrial respiration in C57BL/6 mice with epilepsy (Burtscher et al., 2018). Xu et al. (2020b) showed that DOR activation protected against the MPP^+^-induced disruption of mitochondrial membrane potential and mitochondrial damage, including the production of ROS and the release of cytochrome c, in PC12 cells. A recent study has also found that DOR activation promoted mitophagy and improved mitochondrial function by upregulating transient receptor potential vanilloid subtype 4 in brain microvascular endothelial cells against ischemia/reperfusion injury (Deng et al., 2025).

### Delta-opioid receptor-mediated regulation of microglia

Microglia are macrophage-like immune cells in the CNS, which play a vital role in the inflammatory response of brain diseases, including stroke and neurodegenerative diseases. Activated microglia can be divided into two phenotypes, M1 and M2. The classical M1 activation is linked to cytotoxicity and inflammatory response, while alternative M2 activation is considered beneficial (Spiteri et al., 2022). Our data showed that DOR expression increased in the activated microglial M2 phenotype but slightly decreased in the microglial M1 phenotype (Cheng et al., 2021; Xu et al., 2022). Furthermore, DOR activation promoted microglial polarization towards the M2 phenotype, weakening the occurrence of neuroinflammation (Ryu et al., 2018).

During an inflammatory ischemic process, DOR activation with Delt-D(var) was found to provide ischemic neuroprotection by inhibiting NO production in a lipopolysaccharide (LPS) and interferon-γ (IFN-γ) stimulated N9 microglial cell line (Govindaswami et al., 2008). Activation of DOR with morphine preconditioning alleviated LPS and IFN-γ induced mouse microglial cell injury, as assessed by LDH release, through inhibition of the p38 pathway (Gwak et al., 2010; Ryu et al., 2018). Cheng et al. (2021) found that in LPS-treated BV2 microglial cells, the DOR agonist TAN-67 suppressed the levels of pro-inflammatory cytokines iNOS, IL-1β, and IL-6, and boosted the anti-inflammatory factor IL-10. Moreover, TAN-67 pretreatment attenuated the increase in ERK, c-Jun-N-terminal kinase, p38, and cleaved caspase-3 levels induced by LPS stimulation. These data indicated that DOR-mediated anti-inflammatory and anti-apoptotic are related to the inhibition of MAPK pathways in BV2 microglial cells (Cheng et al., 2021). Our data showed that DOR activation with UFP-512 inhibited LPS and hypoxia-induced microglial M1 phenotype activation. With a high concentration of UFP-512, DOR suppressed the M2 transformation of BV2 cells under IL-4 treatment. Knocking down DOR promoted the excessive activation of BV2 cells in M1 and M2 directions, whereas overexpressing DOR had the opposite effect (Xu et al., 2022). However, MOR activation promoted inflammation through the PKC-Akt-ERK1/2 signaling pathway in LPS-induced microglia (Gessi et al., 2016).

DOR and MOR exhibit distinct modulations of neuroinflammation in microglia during alcohol-induced neurotoxicity (Shrivastava et al., 2017). DOR agonist DPDPE increased the secretion of anti-inflammatory cytokines and suppressed the ethanol-induced increase in inflammatory signaling protein levels, including p38, p-AKT, and NF-κB, and the secretion of pro-inflammatory cytokines in the primary cultured microglia (Shrivastava et al., 2017). On the contrary, MOR agonists aggravated the increase of pro-inflammatory factors and the activation of inflammatory signaling protein triggered by ethanol (Shrivastava et al., 2017). Conversely, MOR agonists worsened the elevation of pro-inflammatory factors and the activation of inflammatory signaling protein induced by ethanol.

DOR activation in microglia protects neuronal cells under various stresses. Under LPS injury or hypoxic stress, the protective effect of DOR on PC12 cells was elevated by co-culture with the medium of BV2 cells treated with DOR agonist (Xu et al., 2022). Shrivastava et al. (2017) showed that adding microglial cell cultures with ethanol and DAMGO to proopiomelanocortin (POMC) cell cultures increased the apoptosis of POMC cells. However, the conditioned medium of microglia with ethanol and DPDPE decreased POMC neuron apoptosis. This finding suggested that DOR is a protective regulator of ethanol-activated microglia killing POMC neurons.

### Mechanisms for delta-opioid receptor protection against ischemic and degenerative injury

Ischemic stroke, AD, and PD are three common diseases in aged people. Their feature is that the number of patients increases with age. At present, there are limitations in the treatment of these diseases in clinical practice. Current evidence suggests that DOR may become a therapeutic target for neurological disorders in the future.

#### Ischemic stroke

**Figure 2:**
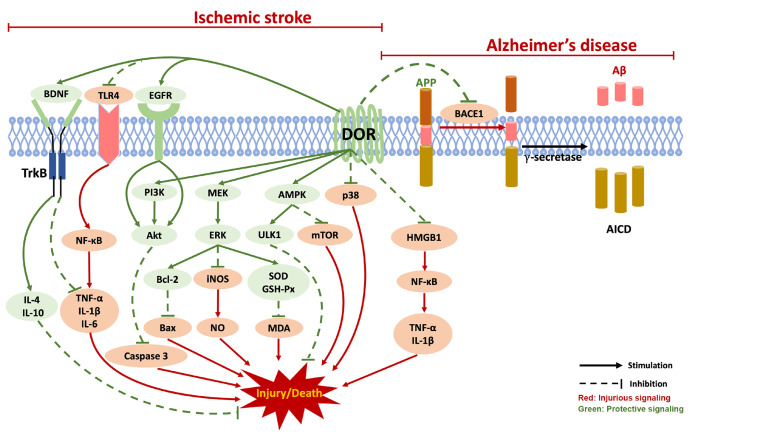
DOR-mediated neuroprotective mechanisms in ischemic stroke and Alzheimer’s disease. The green boxes represent the neuroprotective molecules activated by DOR, and the orange boxes/symbols show the injurious molecules upregulated in the disease conditions. Arrows indicate the increased expression/activity of the downstream molecules and dashed lines represent an inhibitory effect on the downstream molecules. The red arrows represent ischemic/Aβ injury, while the green arrows/dashed lines represent the DOR-induced protective effects through the molecules. Akt: Protein kinase B; AMPK: AMP-activated protein kinase; APP: amyloid precursor protein; Aβ: amyloid-β; BACE1: β-site APP cleaving enzyme 1; Bcl-2: B cell lymphoma 2; BDNF: brain-derived neurotrophic factor; DOR: delta-opioid receptor; EGFR: epidermal growth factor receptor; ERK: extracellular signal-regulated protein kinases; GSH-Px: glutathione peroxidase; HMGB1: high mobility group box 1; IL: interleukin; iNOS: inducible nitric oxide synthase; MDA: malondialdehyde; NF-κB: nuclear transcription factor-kappa B; NO: nitric oxide; PI3K: phosphatidylinositol 3-kinase; SOD: superoxide dismutase; TrkB: tropomyosin-related kinase B; TNF-α: tumor necrosis factor α; TLR4: Toll-like receptor 4.

Ischemic stroke is the second leading cause of death and disability, manifesting as insufficient blood supply caused by cerebral vascular occlusion (Ajoolabady et al., 2021). The multiple signaling pathways implicated in DOR-mediated neuroprotection in various ischemic models have been described previously (Govindaswami et al., 2008; Ke et al., 2009; Yang et al., 2009, 2014; Duan et al., 2011; Wang et al., 2011, 2019; Jeong et al., 2012; Kong et al., 2019; Sun et al., 2020; Chen et al., 2021a; Fu et al., 2021; **Figure 2**).

Growing evidence has indicated that ischemic stroke is a risk factor for AD (Singrang et al., 2024). Furthermore, it has similar pathological features to AD, including changes in amyloid precursor protein (APP) expression, processing, and Aβ production (Ouyang et al., 2021). At 6 hours post-MCAO, Min et al. (2018) found that DOR activation using Tan-67 increased total APP, mature APP levels, and APP processing in the penumbral cortex, while at 24 hours, these levels were decreased. Besides, there is no change in β-site APP cleaving enzyme 1 (BACE1) expression at any time point. In the ischemic core, Tan-67 decreased total APP but not BACE1 expression at 6 hours. Tan-67 increased the total and mature APP levels and suppressed BACE1 upregulation and activity at 24 hours following MCAO. Naltrindole significantly reversed this effect (Min et al., 2018).

#### Alzheimer’s disease

AD is the most common neurodegenerative disorder causing cognitive impairment, which is mainly characterized by amyloid plaques and neurofibrillary tangles, respectively composed of Aβ peptides and hyperphosphorylated tau (Graff-Radford et al., 2021). In our recent study, we proposed that DOR activation has a protective effect on the pathological process of AD (Xu et al., 2020a, 2025). Treatment of DOR agonist UFP-512 significantly decreased BACE1 activity, and naltrindole abolished this effect. However, MOR agonist DAMGO remarkably enhanced BACE1 activity. Hence, DOR and MOR have the opposite effect on BACE1 activity under AD injury (Xu et al., 2020a). We measured the effect of DOR and MOR on Aβ_42_ production. Both DOR inhibition and MOR activation significantly increased Aβ_42_ production. Knocking down DOR increased BACE1 expression and activity and induced more production of Aβ_42_ (Xu et al., 2020a). To further confirm the effect of DOR on BACE1 regulation, we examined APP full length and its cleavage product AICD. UFP-512 remarkably increased the protein expression of APP full length and reduced the level of AICD, suggesting that DOR decreased APP cleavage efficiency (Xu et al., 2020a).

In addition, we found that in both *in vivo* and *in vitro* experiments, DOR and MOR have significant differences in regulating AD-related neuroinflammation and microglial function. In the APP/PS1 mouse model, DOR activation led to a decrease in the release of pro-inflammatory cytokines (TNF-α and IL-1β) in the cortex and hippocampus, while MOR did not have the same regulatory effects on neuroinflammatory responses (Xu et al., 2025). *In vitro* experiments further confirmed that DOR activation also reduced the release of TNF-α and IL-1β in BV2 cells treated with Aβ_1–42_ oligomers (Xu et al., 2025). Mechanism studies have shown that the inhibitory effect of DOR-mediated neuroinflammation is closely related to the inhibition of microglial activation. Subsequently, transcriptome sequencing was performed on BV2 cells treated with Aβ_1–42_ oligomers, and the results showed that overexpression of DOR upregulated microglial homeostasis-related genes (*Cx3cr1*, *Mef2a*, and *P2ry12*) and inhibited disease-related phenotype genes (*Axl*, *Lpl*, and *Tyrobp*). Overexpression of MOR downregulated Cx3cr1 and upregulated Mef2a, Axl, and TMRE (Xu et al., 2025). Based on transcriptome sequencing results, it is speculated that the differential regulation of DOR and MOR on microglia may be related to the regulation of C1q and high mobility group box 1 (HMGB1). Further research found that DOR activation reduced neuroinflammation by inhibiting the nuclear translocation and secretion of HMGB1, blocking its mediated NF-κB signaling pathway. In the co-culture model of neurons and microglia, it was confirmed that the reduced neuroinflammation and cell apoptosis regulated by DOR were achieved by targeting HMGB1 (Xu et al., 2025).

Overall, DOR protects AD against damage and its unique anti-inflammatory and neuroprotective mechanisms in AD suggest that targeting DOR may become a new strategy for AD treatment (**Figure 2**).

#### Parkinson’s disease

**Figure 3:**
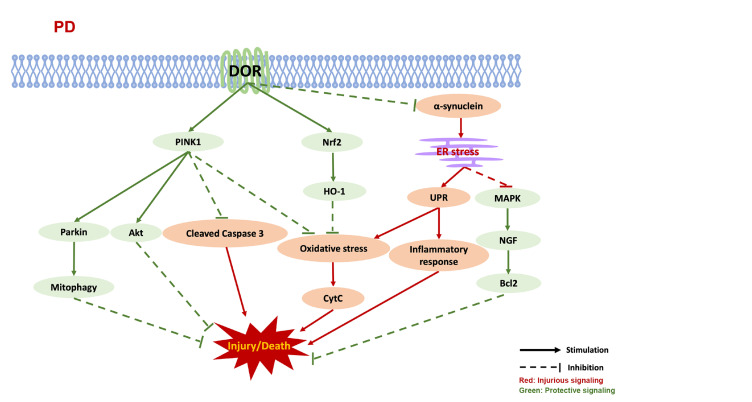
DOR-mediated neuroprotective mechanism in PD. The green boxes represent the neuroprotective molecules activated by DOR, and the orange boxes represent the increased expression/activity of injurious molecules in PD-like (MPP^+^) stress. Arrows indicate the increased expression/activity of the downstream molecules and dashed lines represent an inhibitory effect on the downstream molecules. The red arrow/dashed lines represent injurious effects in PD-like (MPP^+^), while the green arrow/dashed lines represent the neuroprotective effects of DOR. Akt: Protein kinase B; Bcl-2: B cell lymphoma 2; CytC: cytochrome c; DOR: delta-opioid receptor; ER: endoplasmic reticulum; HO-1: heme oxygenase 1; MAPK: mitogen-activated protein kinase; NGF: nerve growth factor; Nrf2: nuclear factor erythroid 2-related factor 2; PD: Parkinson’s disease; PINK1: PTEN-induced kinase1; UPR: unfolded protein response.

The pathology of PD is characterized by the loss of dopaminergic neurons and Lewy bodies in the substantia nigra (Morris et al., 2024). Hypoxic and neurotoxic insults are recognized as underlying causes of PD. The pathological hallmark of PD has been studied in various aspects of mitochondrial dysfunction, oxidative stress, protein misfolding, neuroinflammation, and genetic disorders (Morris et al., 2024). DOR activation is potentially a protective strategy in PD injury through the regulation of PTEN-induced kinase1 (PINK1) and UPR stress (Begum and Sen, 2018; Moghal et al., 2018; Xu et al., 2019, 2020b; **Figure 3**).

*PTEN-induced kinase1 pathway*

PINK1, a mitochondrial-related molecule, is an important protector in nerve cells from Parkinsonism. DOR activation reversed the decrease of PINK1 protein and reduced the increase of caspase 3 in PC12 cells with hypoxia and MPP^+^ injury. Additionally, we found that DOR activation increased the level of phosphorylated Akt under hypoxic/MPP^+^ injury. PINK1 knockdown largely eliminated this effect. It is suggested that DOR-mediated cytoprotection acts by increasing Akt under hypoxic and MPP^+^ injury by mediating PINK1-related signaling pathways in PC12 cells (Xu et al., 2019). DOR activation upregulated PINK1 expression and promoted Parkin’s mitochondrial translocation and modification, thus enhancing the PINK1-Parkin mediated mitophagy assessed by the reduction ratio of mtDNA/nDNA, cytochrome c oxidase subunit II, and two outer mitochondrial membrane protein (Mfn2 and Tom20) under MPP^+^ insult, but not hypoxic injury. Moreover, knocking down PINK1 or DOR disturbed the colocalization of Parkin and mitochondrial translocation under MPP^+^ injury, reconfirming that DOR and PINK1 are essential factors in inducing mitophagy (Xu et al., 2020b).

*Regulation of unfolded protein response stress*

The accumulation of misfolded proteins (α-synuclein) in the ER characterizes the pathological feature of PD, leading to activation of the UPR. UPR stress induced oxidant stress, inflammatory response, and triggered neuronal death (El Manaa et al., 2021). DOR activation with SNC-80 improved motor dysfunction of MPTP-treated mice by reducing UPR stress sensors: IRE-1α/Bip/CHOP, and attenuated inflammatory response, which manifested down-regulation of pro-inflammatory cytokines such as IL-6, TNF-α, IL-1β, and IFN-γ (Begum and Sen, 2018). Furthermore, the improved behavioral response is associated with a reduced oxidant effect after SNC-80 treatment. Similarly, in the PD model of SH-SY5Y cells suffering from ER stress, DADLE-induced DOR activation increased cell viability when treated with ER stress inducers Tm and DTT. The crosstalk between the DOR and MAPK-NGF-Bcl2 pathway enabled DADLE to dampen UPR stress sensors and prevent protein aggregation (Moghal et al., 2018).

## Limitations

Although a large number of *in vitro* and *in vivo* experiments have confirmed the neuroprotective effect of DOR and shown that it is a potential target for preventing and treating neurological disorders, there are still some limitations in current research.

First, most previous studies were performed based on animal and cell models, indicating a need for more clinical research on the use of DOR activation for neurological disorders. It is essential to validate the DOR effect in human research and examine its long-term safety and other impacting factors for clinical use.

Second, more work is needed to define the appropriate dosage for maximal protection. Because of the high homology of opioid receptor structures, opioid receptor agonists, including DOR agonists, have a relative, but not unique, specificity to a particular type of opioid receptor. A high concentration may affect other types of opioid receptors, or even non-opioid receptors. Additionally, the proper dosage might differ depending on physiological/pathophysiological conditions and types of tissue. For example, DOR agonists promote motor function at low doses while inhibiting it at high doses (Mabrouk et al., 2009). The later action is possibly due to a non-DOR effect. When the concentration of DOR agonists increases, off-target effects may occur, thus activating other opioid receptors or nonopioid receptors. Moreover, there are significant variations in the effectiveness of DOR agonists across various cell models. For example, under OGD/R injury, DADLE at 20 μM is the optimal protective concentration in SH-SY5Y cells (Lai et al., 2020), while DADLE at 5 nM exerts neuroprotective effects in brain microvascular endothelial cells (Deng et al., 2025). One of the reasons for this difference is likely due to differential receptor expression and different signaling pathways in different cells. All these issues may affect the outcome effects of DOR agonists on different organs/tissues/cells in various conditions. These issues need to be further explored in future research.

## Conclusions and Perspectives

The use of opioid analgesics has a long history. Recent research has discovered that opioid receptors, especially DOR, have other physiological functions, such as neural protection and regeneration. Our studies and those of others have shown that DOR activation exhibits a significant neuroprotection against hypoxia, ischemia, excitotoxic stress, and neurodegenerative diseases such as AD and PD. Its mechanism covers a multi-level regulatory network, including maintaining ion homeostasis, activating survival signaling pathways, inhibiting apoptosis and necroptosis, regulating autophagy, inhibiting inflammatory responses, and improving mitochondrial function. In addition, DOR plays a significant role in neural generation.

The key findings in this field mainly include the following:

(1) Regulation of ion homeostasis. DOR effectively alleviates K^+^ efflux and Na^+^/Ca^2+^ imbalance caused by hypoxia/ischemia by inhibiting voltage-gated sodium channels, regulating BK channel activity, and antagonizing NMDA receptor-mediated calcium influx, thereby maintaining neuronal membrane stability.

(2) Activation of specific survival pathways. DOR promotes neuronal survival through several signaling pathways, including ERK, PI3K/Akt, and PKC. In addition, DOR enhances antioxidant capacity by activating Nrf2, inhibits the release of pro-inflammatory factors such as TNF-α and IL-6, and forms an anti-inflammatory antioxidant dual protective network.

(3) Inhibition of apoptosis and promotion of autophagy. DOR inhibits pro-apoptotic factors such as caspase 3 and Bax, while promoting autophagy through the AMPK/mTOR pathway. In the PD model, DOR also enhances mitochondrial autophagy and alleviates α-synuclein toxicity through the PINK1/Parkin pathway.

(4) Suppression of neuroinflammation. DOR significantly reduces neuroinflammatory responses by inhibiting the TLR4/NF-κB pathway and promoting polarization of microglia towards the M2 phenotype. In AD, DOR blocks the inflammatory signal mediated by HMGB1 by inhibiting its nuclear translocation, reducing Aβ deposition. And it is in sharp contrast to the function of the MOR.

(5) Regulation of neural regeneration. DOR promotes neural regeneration by improving synaptic morphology, upregulating the expression of neurotrophic factors such as BDNF and STAT, and enhancing neural differentiation.

It is of utmost importance to verify the neuroprotective effects of DOR in human tissues. More research is needed in the future on the pharmacokinetic issues related to DOR agonists, including appropriate dosage in various tissues/cells, the optimal administration method, and the optimization of delivery systems. These studies could offer a new avenue for more effective therapies for neurological disorders.

In summary, a significant body of research supports the positive impact of DOR on neuroprotection and highlights the potential of DOR activation in the treatment of ischemic and degenerative brain conditions.

## Data Availability

*Not applicable*.
